# Non‐Invasive Underwater DNA Sampling Illuminates Red Sea Echinoderm Diversity

**DOI:** 10.1111/1755-0998.70059

**Published:** 2025-10-14

**Authors:** Mai Bonomo, Omri Bronstein

**Affiliations:** ^1^ George S. Wise Faculty of Life Sciences, School of Zoology Tel Aviv University Tel Aviv Israel; ^2^ The Steinhardt Museum of Natural History Tel Aviv University Tel Aviv Israel

**Keywords:** biodiversity assessment, genetic screening, non‐invasive DNA sampling, Red Sea echinoderms, sustainable field methods

## Abstract

Species‐specific non‐invasive underwater DNA sampling remains largely understudied for marine invertebrates despite its potential to revolutionise biodiversity assessment of vulnerable species or fragile ecosystems. Comprehensive species‐specific DNA barcode databases are essential for accurate species identification and taxonomic assignment, particularly at a time of increasingly employed metabarcoding monitoring of marine biodiversity. We present an in situ swab‐based protocol adapted for underwater collection of genetic material, using Red Sea echinoderms as a case study. We sampled 308 individuals from over 50 species across all five echinoderm classes using a newly designed underwater sampling kit applying sterile buccal swabs and an underwater sampling container. The novel sampling protocol was compared to traditional tissue‐based DNA extractions and tested for preservation conditions (fixatives, temperatures and durations). DNA yield from swabs was lower than from traditional tissue biopsies, yet sufficient for all downstream applications. Overall PCR amplification success was 88% (240/274 echinoderm swabs), with a 94% sequencing success rate (202/214), and no significant difference in DNA integrity between swab and tissue methods. Phylogenetic analyses of 231 specimens revealed 37 clades, including 20 novel Red Sea lineages and provisional identifications of cryptic and rare species. Our results demonstrate that underwater swabbing is a rapid (< 1 min per sample), cost‐effective, and non‐destructive, suitable for generating high‐quality genetic data under challenging field conditions. We propose this protocol as an alternative to traditional DNA sampling, providing an efficient approach for studying at‐risk ecosystems and species while prioritising conservation and sustainability and facilitating large‐scale genetic screening of wild populations.

## 1 Introduction

Biodiversity loss is one of the most pressing and impactful consequences of the Anthropocene, largely driven by climate change, habitat destruction and overexploitation (IPCC 2023). The current pace of species extinction, now widely recognised as the ‘sixth mass extinction’, is unprecedented in human history and is driven by accelerating environmental disruption (Ceballos et al. 2015; Díaz et al. 2019; Ceballos and Ehrlich 2023). This rapid decline of biodiversity weakens ecosystem functioning and stability and jeopardises the essential services they provide, including food security, climate regulation and human health, thereby threatening societal stability and quality of life (Díaz et al. 2019; Senior et al. 2024). Yet, efforts to quantify species loss, monitor ecological change and evaluate conservation effectiveness remain severely hampered by a fundamental gap in our baseline understanding of global biodiversity. In particular, the absence of comprehensive taxonomic, ecological and distributional knowledge across vast portions of the biosphere impedes meaningful assessment and management of biodiversity. Central to this problem is the ‘taxonomic bottleneck’—a critical shortage of trained taxonomists and the consequent limitations in accurate species identification, classification and monitoring (Kim and Byrne 2006; Krishna Krishnamurthy and Francis 2012). This constraint not only delays scientific discovery and ecological inference but also compromises evidence‐based conservation at a time when urgent action is imperative.

Over the past decades, molecular methodologies, and particularly DNA barcoding, have emerged as one of the main tools for species identification across the animal kingdom (DeSalle and Goldstein 2019). The development of barcode libraries typically requires the integration of morphological taxonomy with molecular data to accurately assign sequences to species; once these barcodes are established, subsequent species identification can genetically be inferred with little taxonomic expertise. DNA barcoding can also help delineate species where traditional morphology‐based taxonomy cannot, such as with cryptic species or animals at different stages of ontogeny (Layton et al. 2016; Clouse et al. 2005; Borrero‐Pérez et al. 2009). In particular, the mitochondrial gene cytochrome c oxidase subunit 1 (COI) has emerged as the most widely used genetic marker for species delimitation and has proven effective across most known phyla—including marine taxa (Dawnay et al. 2007; Schander and Willassen 2005; Ward et al. 2008; Bronstein et al. 2018). Indeed, COI barcoding has demonstrated high replicability and operational simplicity, offering an objective means for generating baseline biodiversity data, particularly when employed in conjunction with traditional taxonomic, ecological and morphological approaches (Krishna Krishnamurthy and Francis 2012; Zamani et al. 2022). Furthermore, genetic analyses are especially well‐suited for estimating key demographic parameters that provide insights into population responses to landscape alterations and exploitation pressures (Stetz et al. 2011).

Despite its advantages, DNA barcoding necessitates obtaining genetic material directly derived from the target specimen, which normally entails the collection of tissue samples through invasive and damaging methodologies (Taberlet et al. 1999). Moreover, in many cases, target organisms must be physically collected and processed under laboratory conditions to yield sufficient genetic material, a procedure that may necessitate sacrificing the studied specimen. This destructive nature of traditional sample acquisition often entails strict regulatory and public restrictions on sample collection—particularly in sensitive and protected areas or when studying rare and endangered species (Anderson et al. 2024).

In recent years, awareness of the need for more ethical approaches to animal research has increased, driving a rise in publications on non‐invasive sample collection methods (Schilling et al. 2022). Particularly, environmental DNA (eDNA) metabarcoding has emerged as one of the most promising methods for large‐scale, non‐invasive biodiversity monitoring (Valentini et al. 2016; Schenekar et al. 2020). This approach relies on the extraction of genetic material shed by organisms into environmental matrices, such as water, air, or sediment, typically using broad‐spectrum primers targeting conserved loci (such as the mitochondrial COI). The amplified DNA is normally sequenced using high‐throughput platforms and taxonomically assigned to species via reference databases (such as GenBank, BOLD, or MitoFish (Blackman et al. 2022)), which rely on physically collected and taxonomically verified samples. However, these databases are often incomplete, taxonomically biased, and prone to annotation errors, particularly in understudied regions or taxa (Weigand et al. 2019). Therefore, local reference libraries consisting of taxonomically confirmed sequences are essential to improve taxonomic resolution and the accuracy of eDNA‐based methods (Schenekar et al. 2020; Blackman et al. 2022).

The vast majority of non‐invasive genetic sampling methods have been developed for vertebrates, while invertebrates have largely been ignored (Beja‐Pereira et al. 2009; Monroe et al. 2010; Nowland et al. 2015; Schilling et al. 2022). Only 6% of non‐invasive DNA sampling studies to date are estimated to focus on invertebrates (Lefort et al. 2022). Several non‐invasive, species‐specific methods have been developed for terrestrial organisms, such as the collection of hair, faeces and moults, to name a few (Taberlet et al. 1999; Edson et al. 2021). Nonetheless, most of these methods prove impractical or ineffective for marine research, as the difficulty of underwater sampling is exacerbated by the inherent nature of the underwater environment, the organisms themselves, and the limitations posed by snorkelling, SCUBA diving, or the use of remotely operated underwater vehicles (McLeod and Costello 2017; Pak and Leite 2025). As global awareness and conservation efforts of underwater invertebrates grow, so does the necessity for viable non‐invasive sampling methods (Balázs et al. 2020).

Collection and preservation of samples during marine expeditions present additional logistical and regulatory challenges. Sampling must comply with local regulations, which are often stringent regarding the collection of animals or tissue (Dunn et al. 2023). Following collection, specimens must be preserved and transported to laboratories for processing—a process that may span from days to months. This delay from sample collection to processing, coupled with the limited infrastructure available in remote locations, adds to the challenges of maintaining sample integrity (Kilpatrick 2002; Zimmermann et al. 2008; Di Lecce et al. 2022).

## 2 Red Sea Echinoderms as a Case Study

Echinoderms are the largest exclusively marine invertebrate phylum and are a vital part of diverse marine communities, shaping both deep‐sea and shallow water environments (Paine 1974; Lessios et al. 2001; Hermosillo‐Núñez 2019). They are particularly important in coral reef ecosystems, where they control algal proliferation through grazing, maintaining coral health and facilitating coral recruitment when their populations are balanced (Birkeland 1989; Sotelo‐Casas et al. 2016; Clements et al. 2024). Moreover, certain groups of echinoderms also have significant commercial value, driving high demand, which often leads to increased over‐exploitation of wild stocks (Purcell et al. 2023; Watkins et al. 2023; Eriksson and Clarke 2015; Uthicke et al. 2004; Pais et al. 2007).

Despite their ecological significance and the threats they face from commercial exploitation, little is known of the diversity and ecology of echinoderms in many parts of the world, including the Red Sea (Layton et al. 2016; Mahdy et al. 2019). Forming a deep and salty basin stemming from the northwestern Indian Ocean, the Red Sea is considered a hub for endemic species (DiBattista et al. 2015). The scarce taxonomic knowledge and published records on echinoderms from the region predominantly stem from early expeditions and largely rely on morphological species determination (Mortensen 1926; Dollfus and Roman 1981; Clark and Rowe 1971). However, morphological traits alone are often insufficient for delimiting echinoderm species, which may be morphologically simple (Clouse et al. 2005), too rare to sample, or outwardly cryptic. Sea cucumber (Echinodermata: *Holothuroidea*) taxonomy, for example, heavily relies on microscopic skeletal ossicles for classification of otherwise indistinguishable species, and these too have often proven insufficient for correct identification (Kim et al. 2013). Echinoderms are therefore traditionally underrepresented and misidentified in many parts of the world, particularly during field underwater surveys (Lessios et al. 2001; Ward et al. 2008).

Little work has been done on non‐invasive sampling methods in echinoderms, although semi‐destructive methods have been previously demonstrated in selected taxa (Belford 2024). Notably, sea cucumbers have been successfully sampled using buccal swabs for both DNA extraction and sex identification purposes (Nowland et al. 2015; Zixuan et al. 2023). This method of non‐invasive DNA sampling has already proved effective in a range of organisms, such as amphibians (Pidancier et al. 2003), birds (Handel et al. 2006; Vilstrup et al. 2018), reptiles (Koutsokali et al. 2023; Schulte et al. 2011; Lanci et al. 2012), and bovines (Young et al. 2020). Nowland et al. (2015) compared standard protocols for DNA collection from sea cucumbers with buccal and anal swabbing. They found that the swabs yielded higher quality DNA compared to other methods, including tissue biopsies, with no lasting damage to the sea cucumbers. DNA yield from swabs was sufficient for loop‐mediated isothermal amplification (LAMP) in sea cucumbers (Zixuan et al. 2023) and for double digest Restriction Associated DNA sequencing (ddRAD‐Seq) in limpets (Quinteiro et al. 2022). However, these were all performed in sterile laboratory conditions rather than in situ. Balázs et al. (2020) developed a method for swabbing amphibians underwater while SCUBA diving, coupled with an underwater preservation method. This method is based on a commercial swab and patented test‐tube kit, applying a modification that separates sea water from the preservative within the test tube. Although effective, this method is costly and restricted by the need to obtain equipment from a commercial supplier which consequently limits usability.

Here we explore the broad applicability of the swabbing method as a non‐invasive underwater DNA collection method and demonstrate its efficacy in facilitating broad genetic screening of diverse marine invertebrates on coral reefs, focusing on Red Sea echinoderms as a case study. We compare traditional DNA sampling techniques with our cost– and labor–effective in situ swabbing protocol, evaluating yield, purity, and integrity, and estimate the effects of preservation media, storing conditions, and duration of storage prior to DNA extraction. Ultimately, DNA amplification and sequencing were used as the primary proxy for sample usability. We collected swab samples from over 300 specimens, including over fifty echinoderm species of Red Sea origin, and constructed the basic phylogeny of four of the five classes. This constitutes the first molecular data for many of these species in the GOA and greater Red Sea. We illustrate the effectiveness of this swabbing method and propose a pipeline for everyday use as well as during expeditions to remote locations. Given the rapidly deteriorating state of coral reefs globally, we believe this method may pave the way for a new era in marine conservation efforts through the integration of broad‐scale genetic screening of marine communities.

## 3 Methods

### 3.1 Swabbing Kit Design and Deployment

To facilitate underwater sample collection while ensuring cost‐effectiveness, accessibility and reproducibility, we developed a simplified preservation system. This design was adapted from Balázs et al. 2020, which critically separated the mixing of seawater and the preservation solution while underwater.

To reduce costs and enhance accessibility, we evaluated multiple types of collection tubes and ultimately adopted the use of generic 5 mL PET bottles equipped with screwable flip caps. An earlier design, referred to as the ‘double‐tube method’ and shown in Figures S1 and S2, was employed for all samples collected up to January 2024. Prior to use, the PET bottles or tubes were sterilised under UV light for 10 min and subsequently filled to the brim with the chosen preservation buffer—either 100% molecular‐grade ethanol or RNA Save (Biological Industries). A small piece of Parafilm (Amcor) was placed over the bottle opening before securing the flip cap, thereby creating a sealed barrier between the preservation medium and the external environment (see Video S1).

**FIGURE 1:**
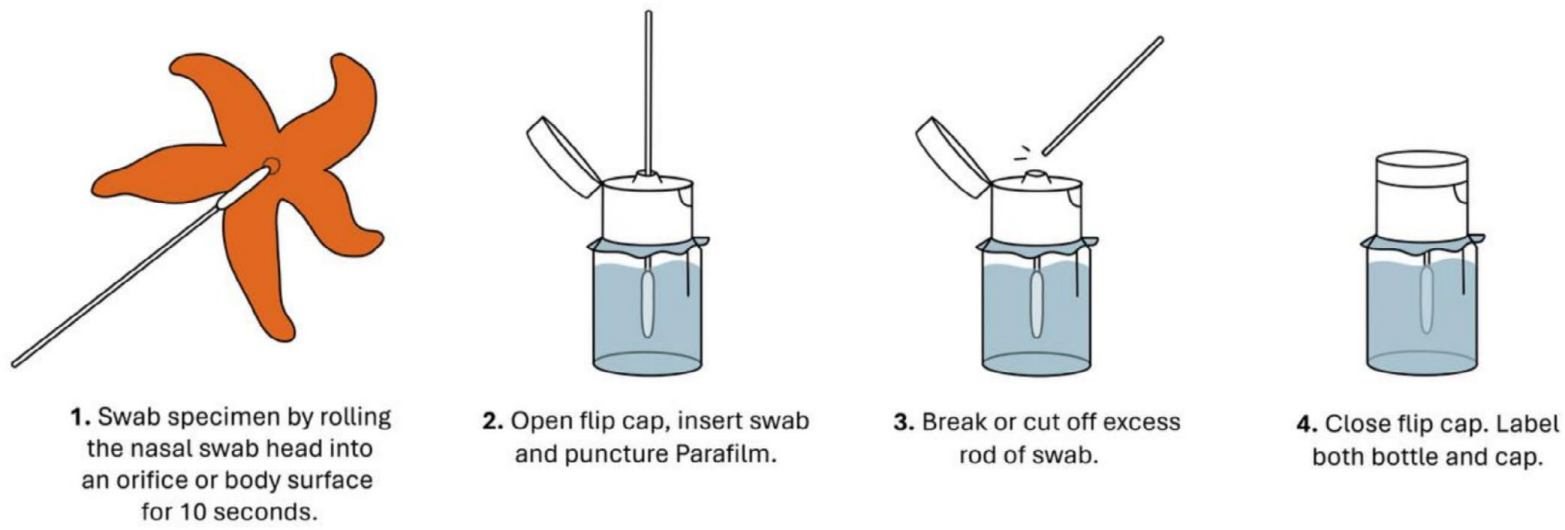
Underwater swabbing workflow.

**FIGURE 2:**
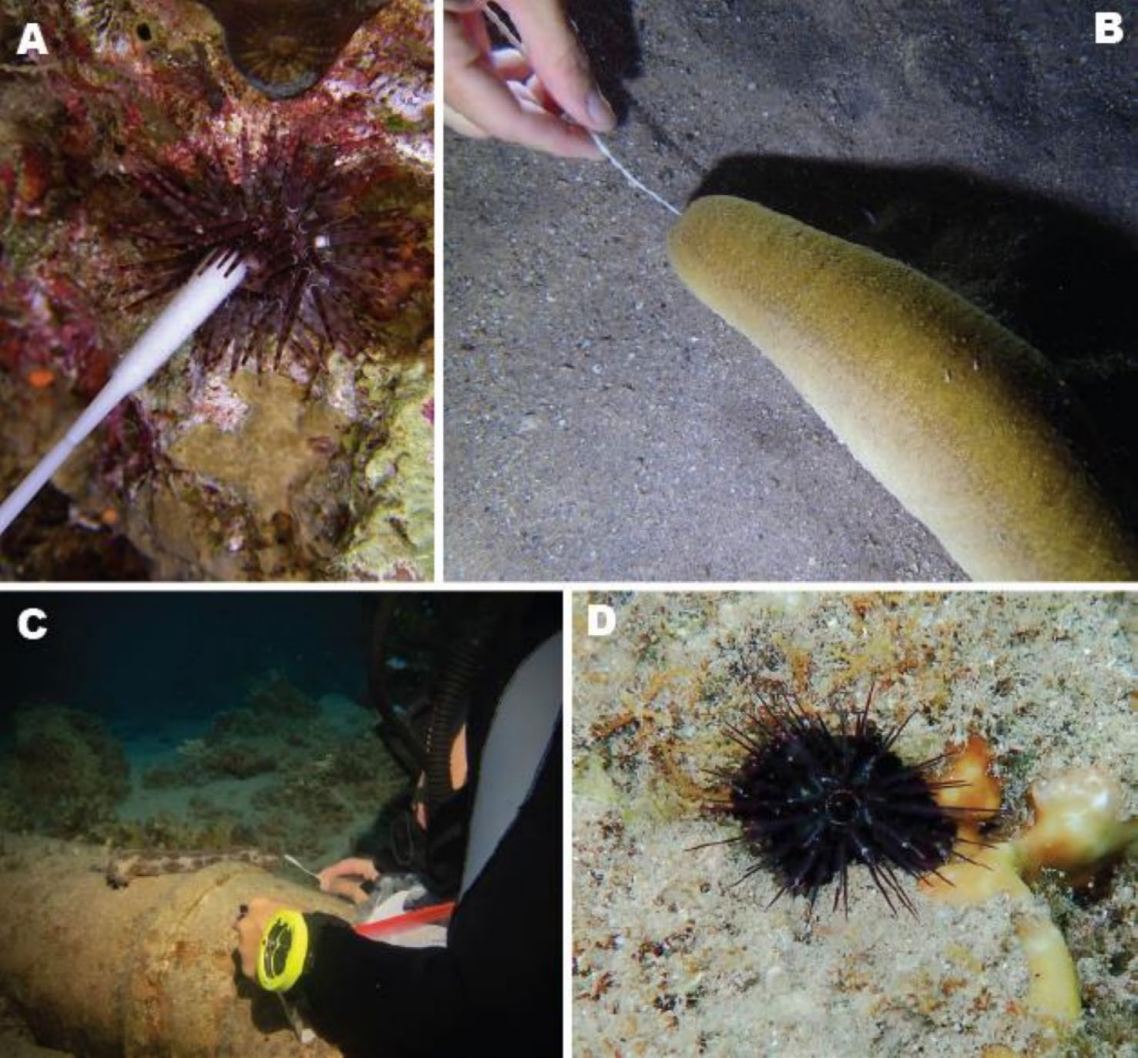
Field photos of echinoderms swabbed in this study. (A) *Parasalenia poehlii* (Courtesy of Julia M. B. Cerutti). (B) *Bohadschia* sp. (C) *Pearsonothuria graeffei* (Courtesy of Shlomi Hai Levy). (D) *Echinostrephus molaris*, a boring sea urchin (Courtesy of Gal Eviatar).

Sampling was done as presented in Figure 1. Specimens were swabbed for 10 s either inside an orifice such as the mouth or anus or on external surfaces, depending on the species, size and accessibility of the target organism (Figure 2). Thereafter, the flip cap of the tube was opened underwater (with the Parafilm layer preventing leakage or mixing of the interior and surrounding aqueous environments), and the swab (head first) was partially pushed in, puncturing the Parafilm. Subsequently, the swab rod was cut at ca. 3 cm from the tip and fully inserted into the tube while being submerged in the preservative. The flip cap was then quickly closed. If done properly, little to no seawater will enter the preservative phase (Video S2).

Each tube was pre‐labelled with a unique numerical identifier. During collection, the species name or, for unidentified specimens, a brief descriptive note was recorded on an underwater slate. Additionally, photographs were taken of the specimen when possible, including one showing the sample tube and number to provide an additional record.

Collected samples were relabeled with unique isolate numbers after the dive and then kept at room temperature, ranging from 18°C to 40°C depending on the collection site, until DNA extraction, which was performed between 1 day and 47 days after collection (see details in Table S1).

### 3.2 Sampling

Underwater sampling was conducted by SCUBA from March 2023 through September 2024. Samples were collected from 19 sites along the Red Sea, with the vast majority being from the Northern Gulf of Aqaba (Table S1). Sampling at the straits of Bab al Mandab (Djibouti) was carried out during the Transnational Red Sea Center (TRSC) Southern Red Sea expedition in November 2023. Additional samples were obtained from native Red Sea fauna reared in land‐based recirculating sea water mesocosm systems at the Interuniversity Institute for Marine Sciences (IUI) and the Underwater Observatory Marine Park in Eilat.

Species identifications were first based on examination of external morphology in situ and comparison with prior knowledge of regional fauna, and subsequently validated by the molecular analyses (see below). In some cases, specimens couldn't be identified to the species or genus level due to lack of sufficient taxonomic data and were given provisional names according to the next possible taxonomic rank. These morphologically based identifications will henceforth be called ‘morphospecies’ in accordance with Sonet et al. (2022).

Apart from echinoderms, several other marine taxa were sampled by swabbing to test for the general applicability of this method. Particularly, several fish samples and eight scleractinian genera (*Asparagopsis, Pocillophora, Stylophora, Sinularia, Platygyra, Acroppora, Hydnophora* and the hydrozoan *Millepora*) were sampled—both during the day, when polyps are normally retracted, and at night when polyps are open and active.

### 3.3 Post Sampling Preservation Comparisons

DNA obtained using swabs was compared to traditional tissue extraction methods in order to evaluate the performance of the new protocol. Using sea urchins from the mesocosm systems at the IUI, 10 specimens—five *Tripneustes gratilla elatensis* and five *Echinometra* sp. EZ (Bronstein and Loya 2013)—were sampled by both methods (i.e., underwater swabbing and tissue biopsy). For the tissue biopsies, approximately five tube feet were utilized from each urchin. Tube feet were collected underwater using tweezers and immediately preserved in 1.5 mL test tubes with 100% ethanol at room temperature until DNA extraction.

In addition, to compare DNA quality under different preservation methods, five individuals of *T. g. elatensis* were swabbed twice. One swab was preserved in RNA Save (Biological Industries) and the other in 100% molecular grade ethanol. Samples were stored for 1 month prior to extractions—six at −20°C, and the remaining four at room temperature (ca. 23°C) (Table 1).

**TABLE 1:** Preservative experiment samples.

Individual	Preservation medium		Conditions	
	RNA save	100% EtOH	−20°C	Room temp
T1	x		x	
		x	x	
T2	x		x	
		x	x	
T3	x		x	
		x	x	
T4	x			x
		x		x
T5	x			x
		x		x

### 3.4 DNA Extraction

DNA was extracted using the GeneAid gSYNC DNA Extraction Kit according to the manufacturer's Animal Tissue protocol, with slight modifications. Briefly, swabs were first transferred to new UV‐sterilised 1.5 mL tubes using a sterile tweezer for each sample, and the lids were left open in a sterile environment for 10 min to allow the ethanol to evaporate. Extractions followed the manufacturer's protocol, with vortex times increased to 10 s where applicable. DNA was eluted in 50 μL of elution buffer, which was pre‐heated to 56°C, and the elution flow‐through was pipetted back into the column and eluted for a second time. Extracted DNA was stored at −20°C.

### 3.5 DNA Yield, Purity and Integrity

Following extraction, DNA purity and yield were analysed using the NanoDrop 1000 Spectrophotometer, as well as the Invitrogen Qubit 4 Fluorometer, applying the Qubit dsDNA Quantification Assay High Sensitivity kit for swab samples and the Broad Range kit for tissue samples. DNA integrity was measured using the Agilent 4200 TapeStation System, applying the Genomic DNA ScreenTape kit.

### 3.6 PCR Amplifications

**FIGURE 3:**
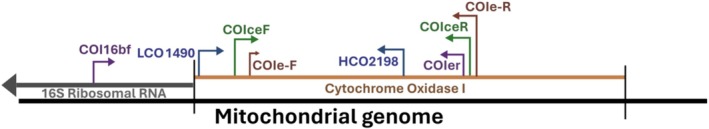
Primer map of the primers used in the present study, focusing on the mitochondrial COI barcode region of echinoderms: LCO1490/HCO2198, COI16bf/COIer, COIe‐F/COIe‐R, COIceF/COIcerR.

The mitochondrial cytochrome c oxidase subunit I (COI) gene was amplified using several primer sets. Echinoderms were amplified with COIceF/COIceR (Hoareau and Boissin 2010), COIe‐F/COIe‐R (Arndt et al. 1996) and COI16bf/COIer (Zeng et al. 2012; Bronstein et al. 2019). Cnidarians were amplified using HCO2198/LCO1490 (Folmer et al. 1994). Fish samples were amplified with three degenerate forward primers, Fish_F1m, Fish_F2m, Fish_F3m and reverse primer Fish_R2m (Tadmor‐Levi et al. 2023). Primer coverage is illustrated in Figure 3, and sequences and PCR conditions are detailed in Table S4.

For all samples, PCR was performed in a total volume of 25 μL, with each reaction mixture containing 12.5 μL of Hylabs Hy‐Taq Ready Mix (2×), 11 μL molecular grade water, 0.25 μL of each primer at 10 μM concentration, and 1 μL of extracted template DNA. All PCR reactions included negative controls. Amplified fragments were visualised on 1%–1.5% agarose gels. Positive amplifications were purified using ExoSAP‐IT and sequenced (in both forward and reverse directions) using the PCR primers at Tel Aviv University's ZABAM sequencing unit or Microsynth GmbH Austria.

### 3.7 Sequence Processing and Phylogenetic Analysis

Chromatograms were reviewed manually, and forward and reverse sequences were compiled into consensus sequences using SeqTrace 0.9.0 (Stucky 2012). Alignments were created per class using AliView version 1.28 (Larsson 2014), implementing MUSCLE (Edgar 2004). In total, 231 COI sequences were deposited in GenBank (accession numbers provided in Table S2).

The generated COI sequences were run through NCBI's nBLAST to infer species assignment or suggest proximate taxa. Comparable COI sequences of species previously reported from the Red Sea, according to the documented distributions in the World Register of Marine Species portal (WoRMS; accessed on 09/09/2024), were downloaded from NCBI GenBank, as well as best match sequences from the BLAST results. These included matches of 98% or higher (Table S2).

Phylogenetic analyses were performed using both Maximum Likelihood (ML) and Bayesian Inference (BI) methods. ML analyses were performed using the IQ‐TREE web server (Trifinopoulos et al. 2016; Nguyen et al. 2014), applying ultrafast bootstrap (UFBoot2; Hoang et al. 2017). Branch support was based on 1000 UFBoot2 replications. Best‐fit substitution models used for each analysis were chosen using ModelFinder (Kalyaanamoorthy et al. 2017) as implemented in IQ‐TREE. PartitionFinder2 (Lanfear et al. 2016; Guindon et al. 2010), implementing the Bayesian Information Criterion (BIC) (Schwarz 1978), was used to identify partitioning schemes and molecular evolution models for the Bayesian analysis, which was then performed using MrBayes 3.2.7 (Ronquist et al. 2012). Two independent runs of three ‘heated’ and one ‘cold’ chain were run for 10 million generations and sampled parameters and trees every 1000 generations. A conservative approach was taken, discarding 25% of the trees as burn‐in, and a 50% majority‐rule consensus tree was calculated on the remaining trees. Bayesian Posterior Probabilities (PP) were obtained from the 50% majority‐rule consensus trees sampled during the stationary phase. Trees were visualised and their topologies compared using FigTree v.1.4.4 (Rambaut 2018).

### 3.8 Statistical Analyses

All statistical analyses were performed using R version 4.4.0 (R Core Team 2024). To compare DNA yield between swab and tissue samples, Welch's two‐sample *t*‐test was performed on the Qubit results, comparing the yield of both sample types from *T. g. elatensis* and *E*. sp. EZ. Welch's *t*‐test was also used to compare the DNA integrity number (DIN) obtained from the TapeStation platform of both sampling methods. A two‐way ANOVA was performed to analyse the effects of temperature and preservation medium on DNA yield.

## 4 Results

In this study, we applied the swab protocol to a total of 308 samples—233 individuals sampled via SCUBA diving or snorkelling, and 75 additional individuals sampled in mesocosms. Sampled species cover a broad range of Red Sea echinoderms from all five classes. Twenty‐one additional samples of corals, sea anemones, and fish were included to test for the broad applicability of the swab protocol.

In the field, the swabbing method proved both easy and efficient while diving, with each sample collected in approximately 1 min, making it possible to collect up to 60 samples for an average 1‐h bottom time dive. Collected samples were contained within a small side‐mounted dive pouch carried by the diver. Each sample was later traceable to species through tube labels, slate notes and photographs.

The sequencing success for all echinoderm samples is summarised in Table S1. Of the 273 echinoderm DNA samples extracted from the swabs, 240 (88%) samples were successfully amplified by PCR. Of these, 214 samples were sent for sequencing (excluding highly redundant species samples), and 202 (94%) were sequenced successfully. Excluding the first few weeks when swabbing and extraction protocols were still being refined and standardised, 227 out of 248 samples (92%) amplified successfully since May 2023, and 195 out of 207 samples (94%) were successfully sequenced.

Of the 16 cnidarians sampled (*Octocorallia, Hexacorallia* and *Hydrozoa*), 14 were successfully amplified (87.5%), and 8 of the 13 samples sent for sequencing were sequenced successfully (61.5%). In addition, a single sea anemone sample was taken after being mistaken for a sea cucumber; this, too, successfully amplified and sequenced. All four fish samples amplified and sequenced successfully.

**FIGURE 4:**
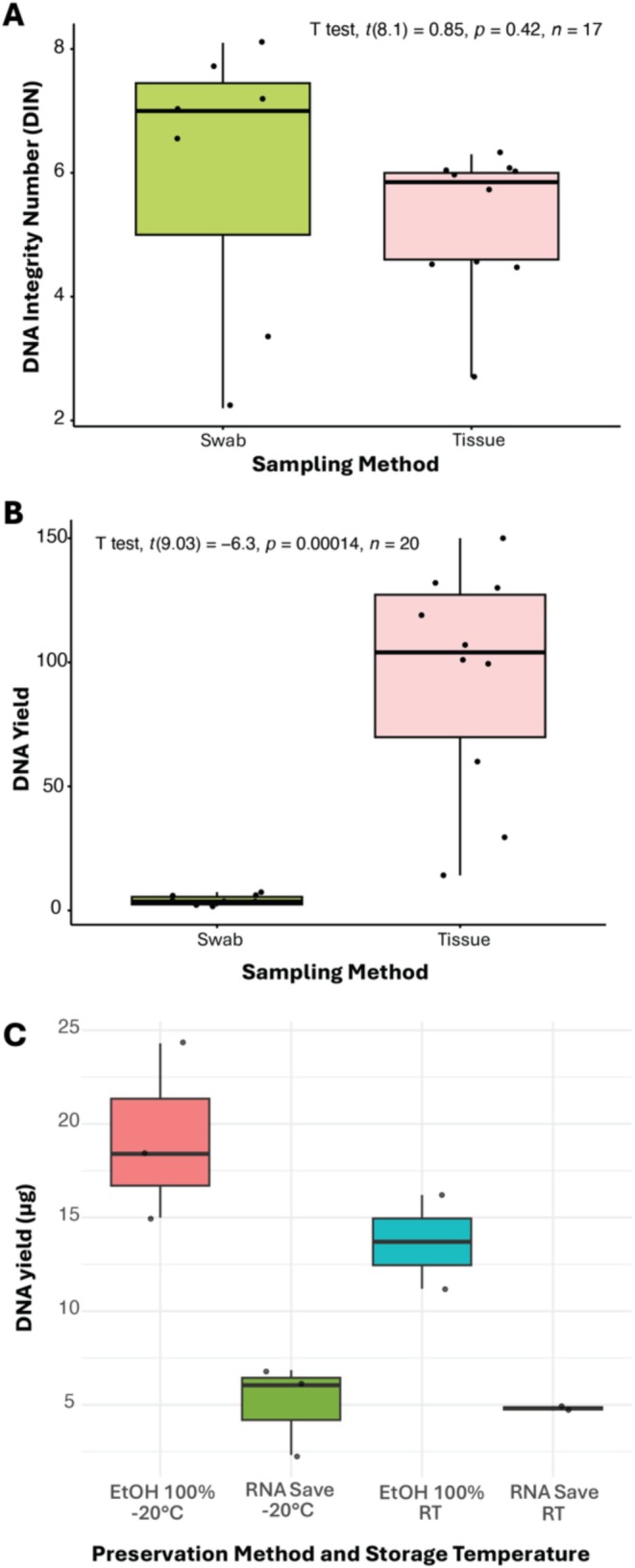
Results of DNA analysis for swabs. (A) Comparison of DNA integrity between swab and tissue samples. (B) Comparison of DNA yield between swab and tissue samples. (C) Comparison of DNA yield between different DNA preservation methods and storage temperatures.

DNA yields of all samples are reported in Table S1. Yields from the swabs ranged between 2.55 and 8400 μg, with a median yield of 293 μg. When comparing DNA yields between the swab and tissue sampling on *T. g. elatensis* and *Echinometra* sp. EZ, the mean yield for swabs was 400 μg, while the mean yield for tissue samples was 9420 μg (Table S5). A Welch's two‐sample *t*‐test showed that this difference was statistically significant (*t*(9.03) = −6.3, *p* < 0.0001, *d* = −2.82) (Figure 4B).

DNA integrity (measured as DIN—a numerical score from 1 to 10 that represents the fragmentation level of genomic DNA on Agilent TapeStation) for these samples was calculated for the same specimens, except for three swab samples that had insufficient DNA concentrations to run the assays (Table S5). The mean DIN was higher for swabs than for tissue—6.03 for swab samples and 5.23 for tissue samples, although this difference was not significant (Welch's two‐sample *t*‐test; *t*(8.1) = 0.85, *p* = 0.42) (Figure 4A).

DNA concentration and DIN values for comparison of storage temperature and preservation medium are reported in Table S6. A two‐way ANOVA revealed no significant interaction between storage temperature and preservation medium (*F*(1, 6) = 1.461, *p* = 0.272). A simple main effects analysis showed that temperature did not have a significant effect on DNA yield (*p* = 0.232); however, preservation medium had a significant effect (*p* = 0.001), with molecular grade ethanol performing better than RNA Save (Figure 4C). Samples were successfully extracted up to 47 days post‐sampling while stored at room temperature (Table S1).

### 4.1 Phylogenetic Analysis

**FIGURE 5:**
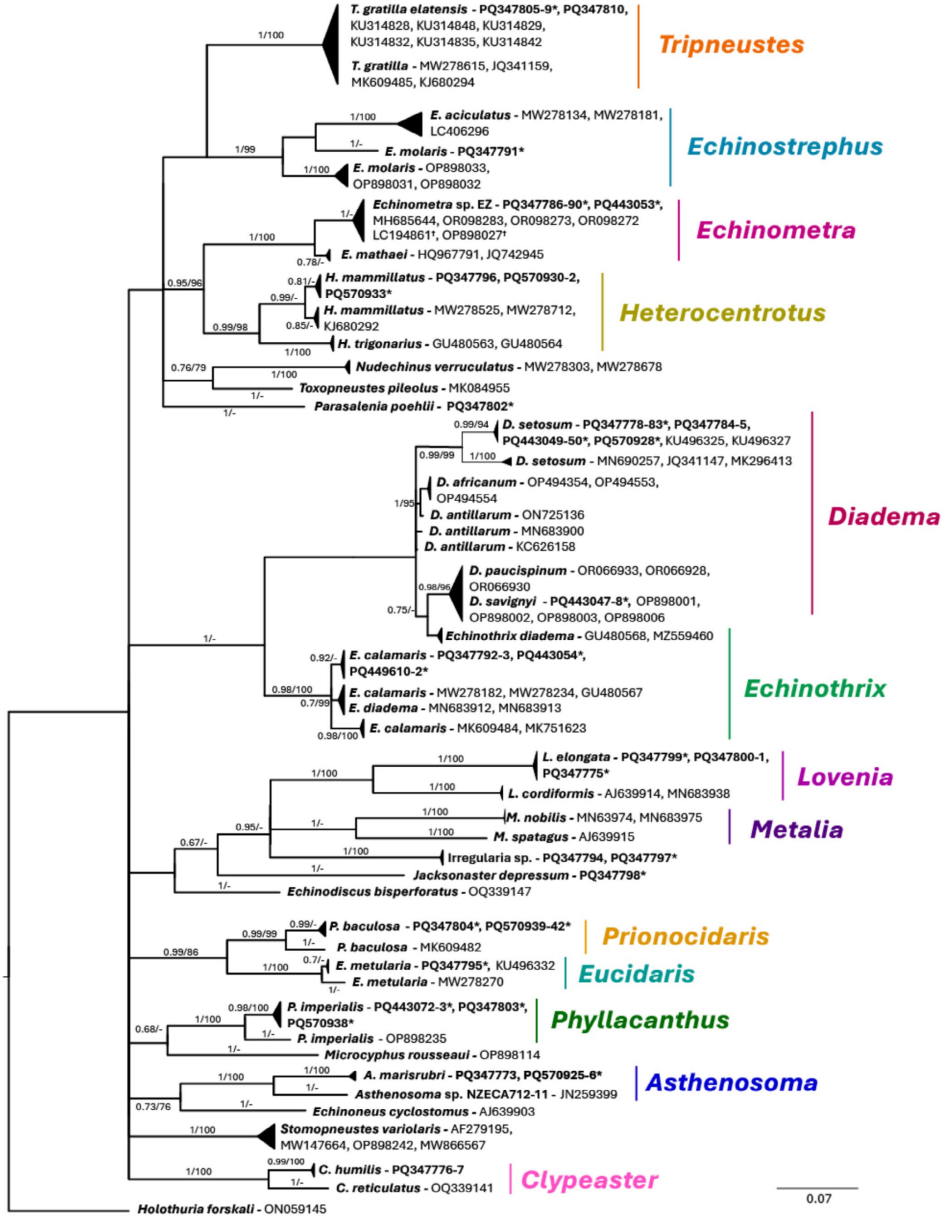
Phylogenetic tree reconstruction of Red Sea echinoids. Bayesian inferred consensus of 75,000 trees generated by 10 million MCMC generations. 138 echinoid sequences were included in the analysis, 57 of these (accession numbers in bold) were generated in the current study. Accession numbers labelled with an asterisk (*) denote sequences from swab samples. Sequences with a dagger (†) denote specimens originally misidentified as *E. mathaei* . *Holothuria forskali* (Echinodermata, Holothuroidea) (GenBank accession number: ON059145) was used as outgroup. The supported > 65 of posterior probabilities of the BI analysis and > 65% of bootstrap replications of the ML analysis are shown above the nodes respectively.

**FIGURE 6:**
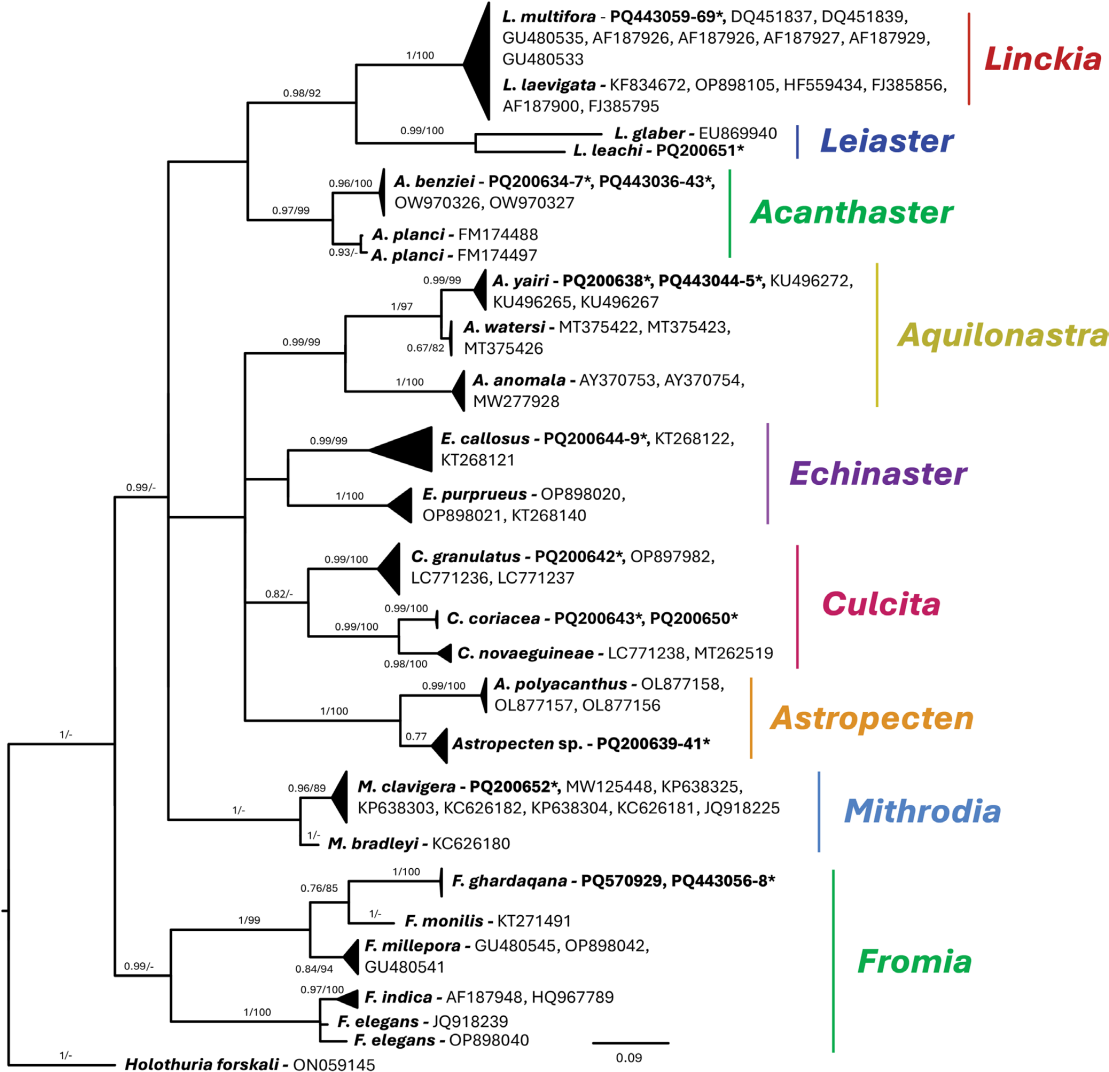
Phylogenetic tree reconstruction of Red Sea asteroids. Bayesian inferred consensus of 75,000 trees generated by 10 million MCMC generations. 100 asteroid sequences were included in the analysis, 44 of these (accession numbers in bold) were generated in the current study. Accession numbers labelled with an asterisk (*) denote sequences from swab samples. *Holothuria forskali* (Echinodermata, Holothuroidea) (GenBank accession number: ON059145) was used as outgroup. The supported > 65 of posterior probabilities of the BI analysis and > 65% of bootstrap replications of the ML analysis are shown above the nodes respectively.

**FIGURE 7:**
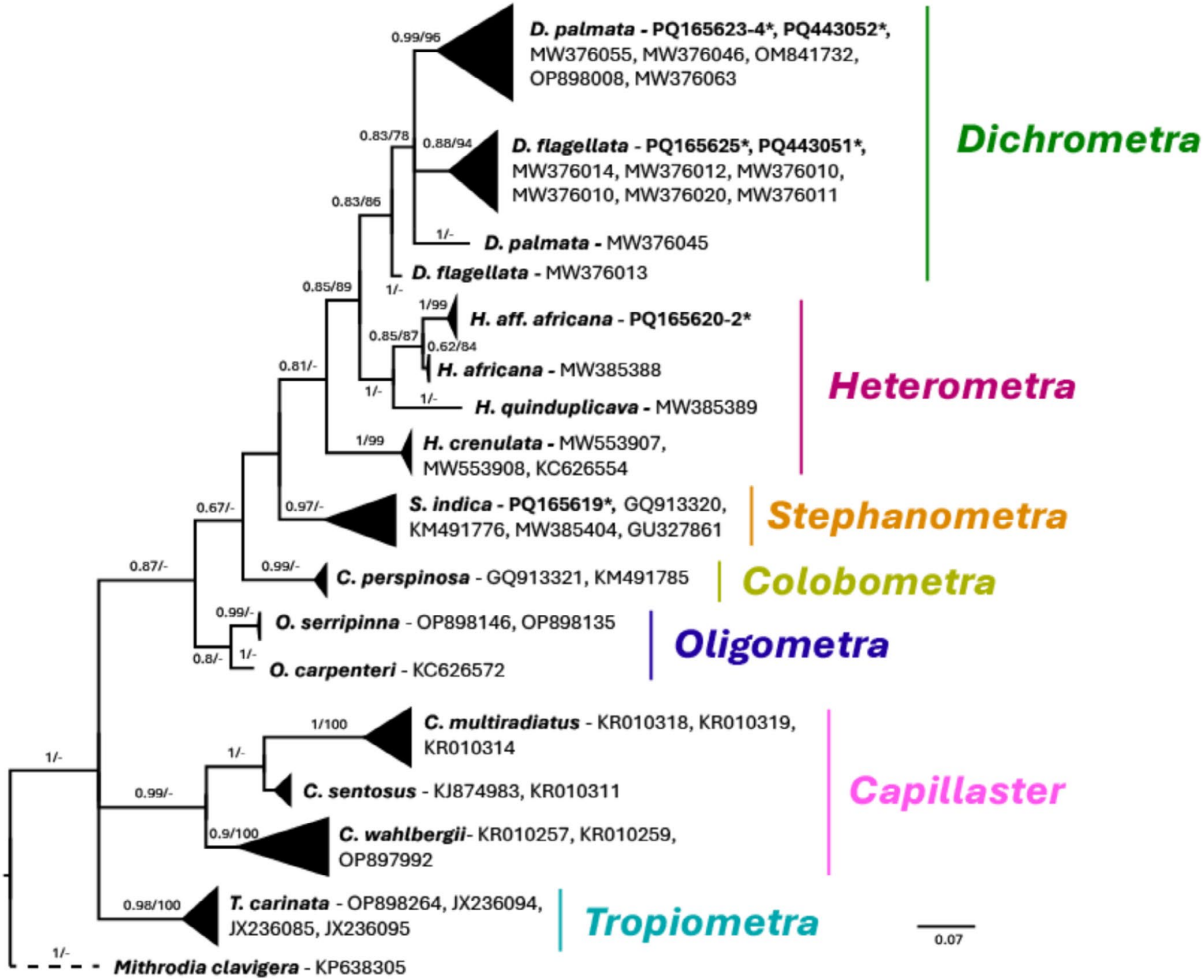
Phylogenetic tree reconstruction of Red Sea crinoids. Bayesian inference phylogenetic reconstruction tree. Bayesian inferred consensus of 75,000 trees generated by 10 million MCMC generations. 49 crinoid sequences were included in the analysis, 9 of these (accession numbers in bold) were generated in the current study. Accession numbers labelled with an asterisk (*) denote sequences from swab samples. *Mithrodia clavigera* (Echinodermata, Asteroidea) (GenBank accession number: KP638305) was used as outgroup. The supported > 65 of posterior probabilities of the BI analysis and > 65% of bootstrap replications of the ML analysis are shown above the nodes respectively.

**FIGURE 8:**
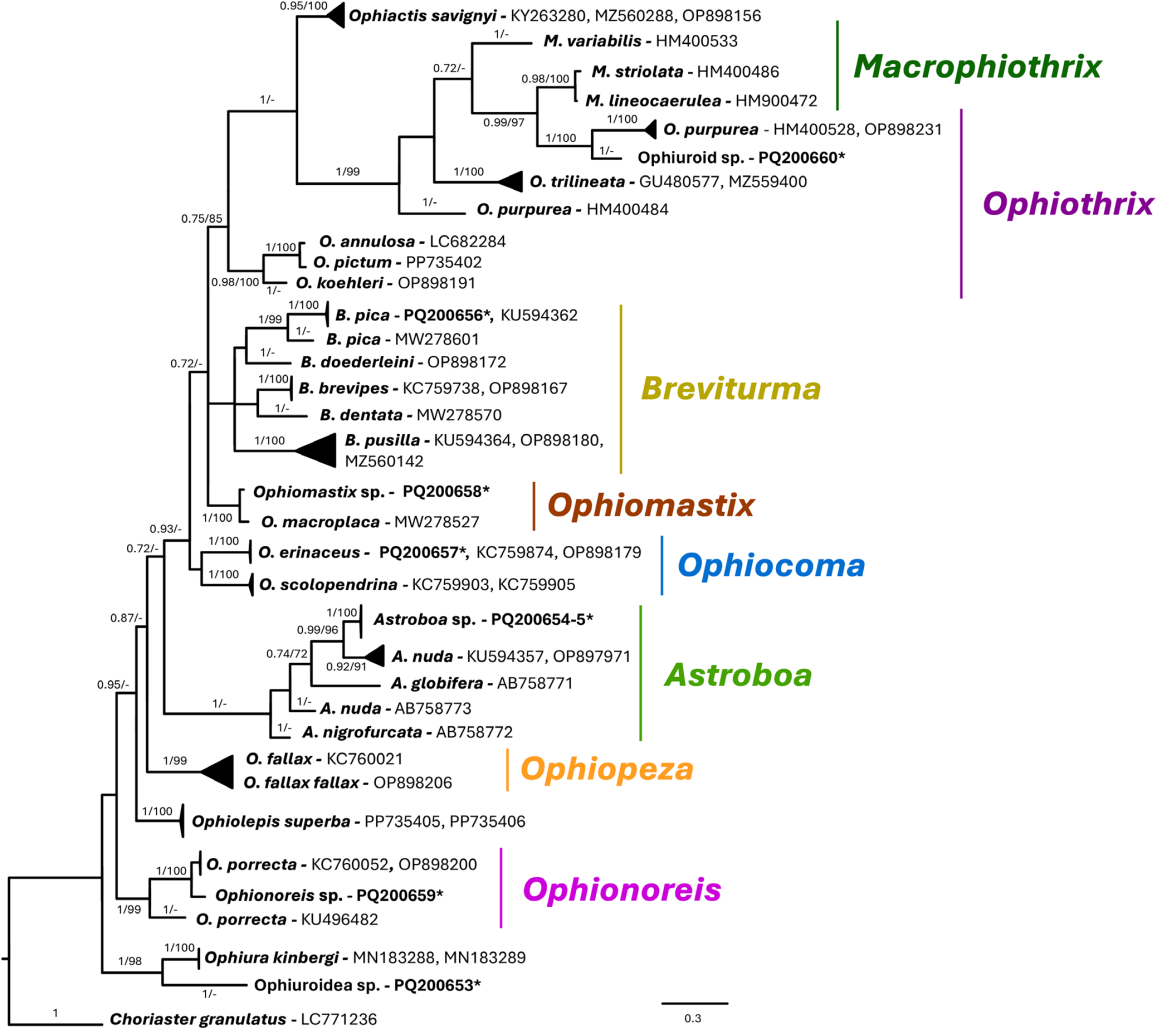
Phylogenetic tree reconstruction of Red Sea ophiuroids. Bayesian inference phylogenetic reconstruction tree. Bayesian inferred consensus of 75,000 trees generated by 10 million MCMC generations. 51 ophiuroid sequences were included in the analysis, 8 of these (accession numbers in bold) were generated in the current study. Accession numbers labelled with an asterisk (*) denote sequences from swab samples. *Choriaster granulatus* (Echinodermata, Asteroidea) (GenBank accession number: LC771236) was used as outgroup. The supported > 65 of posterior probabilities of the BI analysis and > 65% of bootstrap replications of the ML analysis are shown above the nodes respectively.

Phylogenetic trees were constructed independently for four of the five echinoderm classes—Echinoidea (495 bp), Crinoidea (446 bp), Asteroidea (432 bp) and Ophiuroidea (490 bp) (Figures 5, 6, 7, 8). Although holothuroids constituted the majority of sampled individuals, only PCR amplification and sequencing success were evaluated in the current study (Table S1); detailed phylogenetic and diversity analyses for this class are the subject of a forthcoming publication. As BI‐inferred topologies were similar to the ML topologies, only Bayesian trees are shown with both posterior probabilities and bootstrap support values plotted next to their respective nodes.

Across the resulting trees, the Red Sea specimens clustered into 37 distinct clades. Of these, our sequence data revealed 20 unique clades composed entirely of individuals collected during the present study, with no comparable publicly available data. Provisional species names were assigned to either clade with low support (i.e., those containing only one other sequence or where morphological confirmation was not possible) or to unique, unidentified clades.

Most morphospecies were correctly identified in situ and clustered within clades corresponding to or closely related to the identified species. Other morphospecies were classified as unknown during field sampling, or were mistakenly identified, and were only identified post‐BLAST and phylogenetic analysis. Unidentified morphospecies were named based on genetic clustering within the phylogenetic trees and a BLAST match threshold of above 98%.

### 4.2 Echinoidea

Most echinoid morphospecies were correctly identified in situ as confirmed through BLAST searches, based on our own taxonomic expertise with this group, with the exception of two specimens of a single irregular echinoid (*Irregularia* sp. (accession no. PQ347794, PQ347797), sister to *Metalia* and *Lovenia*) (Figure 5). Three morphospecies clustered with confirmed GenBank sequences of the same species (*Diadema setosum*, *Eucidaris metularia, Diadema savignyi*), while five others formed clades that were unique to the Red Sea but sister to their identified morphospecies (*Echinothrix calamaris, Heterocentrotus mamillatus, Prionocidaris baculosa, Phyllacanthus imperialis, Echinostrephus molaris*). Six additional morphospecies (*Lovenia elongata*, *Parasalenia poehlii*, *Irregularia* sp., *Jacksonaster depressum*, *Asthenosoma marisrubri* and *Clypeaster humilis*) formed unique clades with no comparable COI sequences available on GenBank.

### 4.3 Asteroidea

All asteroid morphospecies, with the exception of *Astropecten* sp., were correctly identified to the species or genus level in situ based on our own regional taxonomic expertise and later confirmed genetically. Five morphospecies clustered with confirmed GenBank sequences. These included the following, together with their respective accession numbers: *Acanthaster benziei* (PQ200634‐PQ200637, PQ443036‐PQ443043), *Mithrodia clavigera* (PQ200652), *Aquilonastra yairi* (PQ200638, PQ443044‐PQ443045), *Choriaster granulatus* (PQ200642) and *Echinaster callosus* (PQ200644–PQ200649) (Figure 6). Four morphospecies ( *Leiaster leachi* (PQ200551), *Culcita coriacea* (PQ200643, PQ200650), *Astropecten* sp. (PQ200639–PQ200641) and *Fromia ghardaqana* (PQ570929, PQ443056–PQ443058)) formed unique clades without comparable published sequences, but were placed within the known respective genera. One morphospecies, *Linckia multifora* (PQ443059‐PQ443069), identified morphologically, fell into a polyphyletic clade alongside *Linckia laevigata* and other *L. multifora* sequences.

### 4.4 Crinoidea

Most crinoid morphospecies were misidentified or unidentified in situ, and their names were subsequently revised based on DNA sequences and corresponding BLAST results (Figure 7). Species names were assigned according to confidence level: sequences with > 98% coverage to reliably identified and published references were designated at the species level. For example, sample 21C (PQ165625) was assigned *Dichrometra flagellata* after being misidentified originally as *D. palmata* . Species with reliable references but coverage between 95% and 98% were given provisional names due to uncertainty, such as *Heterometra* aff. *africana*. In the case of this morphospecies, two specimens, 14C (PQ165620) and 16C_20.6.23 (PQ165623) were originally identified as *Heterometra savignii*, and a third (11C, or PQ165621) was originally identified as *Capillaster multiradiatus*.

### 4.5 Ophiuroidea

None of the ophiuroid morphospecies could be unambiguously identified in situ, with the exception of *Astroboa*, which is readily distinguishable as a large basket star. Instead, identification was assigned based on DNA sequences and BLAST results. Two morphospecies (*Breviturma pica* and *Ophiocoma erinaceus*) clustered with confirmed sequences of the same species with high support values (Figure 8) and had strong BLAST support (99.08% and 99.85% identity, respectively) with taxonomic reliability. Four morphospecies had either low BLAST percent coverage, unreliable taxonomic assignment, and formed unique clades in the phylogenetic analysis, and were therefore assigned to the genus level alone (*Astroboa* sp., *Ophiomastix* sp., *Ophionoreis* sp., *Ophiothrix* sp.). One specimen, 8OP (accession no. PQ200653), did not cluster with any genus and had no comparable BLAST results, and was therefore assigned the general *Ophiuroidea* sp.

## 5 Discussion

### 5.1 The Swabbing Protocol

The underwater swabbing method proved highly effective. One of the key advantages is in its ease of use and non‐invasive nature, which is particularly important for studying sensitive environments like coral reefs. As coral reef environments are threatened globally (Riegl et al. 2009), and marine fauna are facing increasing pressures from human exploitation, developing scientific methods that minimise harm to already vulnerable ecosystems should be a priority for both scientists and practitioners. Traditional sampling methods that necessitate tissue biopsies can be highly destructive and take a high toll on organisms that may already be experiencing increased stress. For example, in the GOA, destructive sampling techniques on coral reefs are largely prohibited and local regulations restrict sample collection, consequently delaying taxonomic inventories. This shortcoming is perhaps most profound in population genetics studies, where a large sample size is required to adequately represent genetic diversity (Nazareno et al. 2017; Qu et al. 2019); thus, the cumulative stress on target species can be substantial. With the current procedure, a large number of samples can easily be collected with negligible environmental consequences.

While eDNA sampling and metabarcoding are gaining popularity, particularly due to their non‐invasive nature, scalability, and ease of sample collection, their reliability and accuracy are predominantly reliant on individually collected DNA sequences compiled in public databases. However, publicly available sequence references are often geographically biased, with the majority of entries coming from well‐studied regions in North America and Europe, while vast areas—particularly in the tropics and parts of the Southern Hemisphere—remain underrepresented. This imbalance can lead to misidentifications or coarse taxonomic assignments in global eDNA and metabarcoding studies, reducing the accuracy of biodiversity assessments (Weigand et al. 2019; Marques et al. 2021). As a result, researchers often supplement eDNA studies with physical sampling (Schenekar et al. 2020; Blackman et al. 2022). In this context, swabbing emerges as a promising tool for non‐lethal, species‐specific DNA sampling that can complement eDNA surveys and enable the generation of high‐quality genetic references for taxa and regions of interest.

Non‐invasive and sustainable sampling methods are indeed becoming more prevalent in wildlife research, although there is still notable disparity in attention between terrestrial and marine environments, particularly in invertebrates (Beja‐Pereira et al. 2009; Schilling et al. 2022). The swabbing method demonstrates its versatility for both vertebrates and invertebrates in a variety of underwater environments and during long‐term field expeditions, as demonstrated by the samples collected during the TRSC Southern Red Sea Expedition in 2024. We found that swabs were effective in collecting high‐quality DNA not only from mucus‐secreting animals such as corals, but also from tissues and inner cavities of markedly diverse organisms such as the five echinoderm classes, demonstrating the applicability of this method for a wide range of marine taxa. Although sampled organisms may experience brief stress during swabbing, no lasting harm to either the organism or its surrounding habitat was noted (based on long‐term continuous monitoring of mesocosm individuals).

Sample collection using swabs is rapid, with each sample collected in under a minute. During field work, the limiting factor was time spent locating the organisms, rather than the sampling process itself. Night sampling, while requiring additional instrumentation (lighting), can be managed effectively with the use of hands‐free flashlights or assistance from a dive buddy. The method can easily be adapted to a range of challenging or environment‐specific conditions. One example of the robustness of this method is the samples collected during the TRSC expedition, where ambient temperatures exceeded 40°C, with collected samples remaining viable for over 47 days without refrigeration. Its reliability under such harsh field conditions makes it an ideal choice for prolonged expeditions or research conducted in remote areas where laboratory infrastructure is limited.

Additionally, the collection tubes developed in this study are made from inexpensive, readily available materials, making the method easily implemented within the constantly growing number of citizen scientist projects. One of the key benefits of the swab kit design is the utilisation of liquid‐filled collection tubes (pre‐filled with the preservative)—this in turn reduces their buoyancy and enables easy operation underwater. Furthermore, in contrast to commercial ‘dry’ (air‐filled) collection tubes, the liquid phase separation design eliminates pressure differences between the sealed preservative and surrounding aqueous environment—facilitating easy operation at depth—where commercial dry tubes fail to open due to sub‐pressure forming within the tubes in water as shallow as 10 m.

### 5.2 DNA Extraction and Preservation

Although various methods can be used to extract DNA from swabs (Nowland et al. 2015), we opted for a commercial kit that is both widely accessible and cost‐effective. As expected, DNA yields from swabs were lower than those from tissue biopsies, although the former still provided sufficient DNA of high integrity and purity to allow for successful amplification and sequencing. With respect to preservation solution, molecular‐grade ethanol, even at room temperature, was more than sufficient for the majority of swab samples and largely outperformed the commercial RNA Save (Figure 4C). While ethanol promotes precipitation and stabilization of DNA, the reduced efficiency of RNA Save may be due to its inherent design to stabilize RNA by inactivating RNases and preserving RNA integrity, rather than preserving high molecular weight double‐stranded DNA. This finding is particularly important for field researchers, as ethanol provides a cost‐effective, easily available preservative that doesn't require complex storage conditions.

In samples that failed to amplify or sequence, low DNA yield and the presence of PCR inhibitors are potential sources (Table S1). This may have resulted from swabbing areas with lower tissue content, swabbing too briefly, or targeting regions with a higher concentration of DNA inhibitors, such as the highly pigmented epidermis of sea urchins like *Diadema setosum* or *Echinothrix calamaris*. Using a DNA extraction kit specifically designed for low‐yield samples or employing DNA purification techniques such as magnetic beads separation is likely to improve amplification success.

### 5.3 The Case Study of Red Sea Echinoderms

The Red Sea, with its unique characteristics and high levels of endemism, offers an excellent opportunity to explore molecular distinctiveness in local echinoderm populations. In several cases, specimens that were morphologically similar to known species, such as *Echinostrephus molaris* and *Phyllacanthus imperialis*, formed distinct Red Sea clades (Figure 5). This may be the consequence of geographic isolation, as expected in edge‐of‐range populations (Hardie and Hutchings 2010), such as the GOA. The presence of unique clades warrants further taxonomic analysis and could potentially reveal new endemic species and overlooked species diversity, or reflect potential species misidentifications, further underscoring the need for integrative taxonomic methods (Sonet et al. 2022).

#### 5.3.1 Echinoids

All regular echinoid species encountered during the present study were identified in situ and later confirmed genetically using the swab method. The taxonomically more challenging irregular echinoids, many of which were unidentifiable in the field, were similarly successfully resolved using swab sampling. Several echinoid species encountered during the field surveys provide interesting examples for the advantages of the swab method. One such example is *Echinostrephus molaris* (accession number PQ347791; Figure 2D), a boring sea urchin that lives embedded within rock or coral and had only been observed once in the GOA more than half a century ago (Lawrence 1970). Traditional tissue sampling techniques for this species can be damaging, not only to the urchin itself but also to the coral and surrounding fauna. However, using the swab method we were able to collect sufficient genetic material without causing harm to the urchin or coral host, to confirm the observations of Lawrence (1970), and provide the first genetic reference for this rare species in the region. Interestingly, the genetic data obtained in the current study positions *E. molaris* from the GOA in a separate clade from the species Indo‐Pacific populations and as sister to *E. aciculatus* (Figure 5)—suggesting the presence of currently undescribed diversity. Another noteworthy example is *Parasalenia poehlii* (accession number PQ347802; Figure 2C), the first specimen of its kind to be directly reported from the GOA in decades (Clark and Rowe 1971). Instead of risking harm by removing or stressing the specimen, we were able to document it and collect the first genetic material for this species, further illustrating the method's suitability for studying rare or hard‐to‐access species.

The Strait of Bab al Mandab, which connects the Red Sea to the Indian Ocean, serves as a biogeographical barrier, shaping the evolution and distribution of Red Sea endemism (DiBattista et al. 2015). However, sampling at this location is challenging given its remote location, harsh environmental conditions, and strict regulation on collecting specimens. Nevertheless, to elucidate the biogeography and endemism of Red Sea fauna, obtaining samples from this location is crucial. For example, using swabs, we established that *Diadema savignyi*, a common species in the Indian Ocean (Bronstein and Loya 2014), is present at Bab al Mandab (Figure 5) but absent from the northern GOA, and that *Echinothrix calamaris* across the entire Red Sea—from the GOA to Djibouti—forms a unique genetic clade, distinct from its Indo‐Pacific populations.

#### 5.3.2 Asteroids

Several asteroid species yielded sequences that, to the best of our knowledge, are the first available for their respective species. These include *Leiaster leachi* , *Culcita coriacea* , *Fromia ghardaqana* , and an unidentified *Astropecten* sp. The latter, from a genus known for its taxonomic complexity (Zulliger and Lessios 2010), remains cryptic and unresolved, mainly due to the relatively large number of congenerics in the Red Sea (Zulliger and Lessios 2010).

One particularly interesting species is *Acanthaster benziei*, recently described as a distinct species within the crown‐of‐thorns (COTS) species complex, endemic to the Red Sea (Wörheide et al. 2022) and long misidentified in the region as the infamous coral predator *Acanthaster planci* (Uthicke et al. 2023). The original samples in the Wörheide study were taken from the central Red Sea along the Saudi Arabian coast. Thus, the present swab samples obtained from across the entire stretch of the Red Sea—from the northernmost part of the GOA to the straits of Bab al Mandab confirm the presence of *A. benziei* across the entire Red Sea. Another case worth noting is *Linckia multifora* , whose COI sequences formed a polyphyletic clade with *Linckia laevigata* (figure 9). Despite clear morphological differences between the two (Williams 2000), their sequences are nearly indiscernible (over 98% sequence identity), making molecular identification based on COI alone unreliable for these species (Sonet et al. 2022; Williams 2000; Crawford and Crawford 2007). This finding underscores the limitations of relying solely on COI barcoding for species delineation, particularly in cases where morphological and molecular data may not always align. Nevertheless, using the swab method, researchers can significantly increase sampling efforts in order to capture the true scale of species diversity.

#### 5.3.3 Crinoidea and Ophiuroidea

Crinoids and ophiuroids presented unique challenges in this study due to the limited taxonomic data available for the region and the cryptic nature of these groups. Most crinoids could not have been unambiguously identified to species level in situ, and the majority of ophiuroids were sampled randomly with, at most, genus‐level identification. As a result, the names assigned to these taxa in the current study are based entirely on the DNA barcoding results obtained through swabs.

Crinoids also exhibit a wide range of intraspecific variation, making field identification challenging even for experts (Roux et al. 2013). Furthermore, crinoid phylogeny is complex and relationships are continually being updated, with new species regularly described (Rouse et al. 2013). Thus, genetic data allowed for assigning species names with more confidence than would have been possible through visual identification alone. However, barcoding alone is not without its drawbacks. In cases where morphological variation is not well documented or molecular reference data are incomplete, barcoding may lead to misinterpretations or the clustering of cryptic species into unresolved clades (Cheng et al. 2023; Allison et al. 2024). In the current study, three crinoid sequences (PQ165620‐2) clustered together with *Heterometra africana* (Figure 7), which has not yet been recorded in the Red Sea (WoRMS 2024). Clearly, further morphological examination of these specimens is required to confirm the genetic determinations.

Similarly, ophiuroids are known for their high species diversity and cryptic nature (Stöhr et al. 2009, 2020). The random sampling strategy employed in the current study yielded a diverse range of taxa, ranging from *Ophiothrix* to *Astroboa* (Figure 8), for which no former knowledge is available from the region. An easily recognisable species in situ was *Astroboa nuda*, a large basket star that is common in the northern GOA (Clark and Rowe 1971; Tsurnamal and Marder 1966; Webb et al. 2021). However, our phylogenetic results point to a strongly supported separation between GOA specimens and other publicly available sequences of *A. nuda* (Figure 8), none of which are from the Red Sea. This may represent another example of genetic structuring unique to the Red Sea.

## 6 Conclusions

The present study demonstrates the effectiveness and versatility of non‐invasive DNA sampling using swabs across a variety of marine species and regions, focusing on the unique ecosystems of the Red Sea. By applying this method to groups like echinoderms, which often present identification challenges due to cryptic species or inaccessibility, we were able to successfully collect high‐quality DNA without harming the organisms or their fragile coral reef habitats (Bruckner and Dempsey 2015). The results highlight the potential of this method to become a critical tool for biodiversity studies, especially in regions where conservation and minimising environmental impact are paramount. Many of the new barcodes thus represent the first important step towards cataloguing the echinoderm diversity in the GOA, laying the foundations for an integrative approach—combining molecular, morphological, and ecological data—that will be necessary to fully understand the diversity and distribution of Red Sea echinoderms.

The high levels of endemism and unique genetic signatures within this region highlight the importance of swabbing as a sustainable technique for cataloguing biodiversity by conducting genetic screening in underexplored areas. These findings call for the refinement of traditional methods, combining non‐invasive genetic procedures, to keep pace with current ecological challenges and technological advancements. As conservation efforts and biodiversity studies continue to evolve, it is time to update the scientific toolbox to better align with sustainability goals while delivering deeper insights into species diversity.

## Author Contributions

M.B. and O.B. designed the study and analyzed the data. M.B. collected the data and led the writing of the main manuscript draft. O.B. supervised and guided the project and acquired the funding.

## Disclosure

Benefits Generated: Benefits from this research accrue from the sharing of our data and results on public databases as described above.

## Conflicts of Interest

The authors declare no conflicts of interest.

## Supporting information


**Data S1:** men70059‐sup‐0001‐Supinfo.docx.

**Table S1:** men70059‐sup‐0002‐TableS1.xlsx.

**Table S2:** men70059‐sup‐0003‐TableS2.xlsx.

**Table S7:** men70059‐sup‐0004‐TableS7.xlsx.

**Video S1:** men70059‐sup‐0005‐VideoS1.MOV.

**Video S2:** men70059‐sup‐0006‐VideoS2.mov.

## Data Availability

Unique haplotype data are deposited to NCBI Nucleotide Database under GenBank accession numbers PQ165619–PQ165625, PQ200634–PQ200660, PQ281811, PQ347773–PQ347810, PQ443036–PQ443073, PQ449610–PQ449612, PQ570925–PQ570926, PQ570928–PQ570933, PQ570938–PQ570942. Additional metadata is provided in the main text and Tables S1 and S2.
