# Supplementary material for: Non‐Invasive Underwater DNA Sampling Illuminates Red Sea Echinoderm Diversity
Source: Mol Ecol Resour. 2025 Oct 14;26(1):e70059. doi: 10.1111/1755-0998.70059 (PMC12627914; doi:10.1111/1755-0998.70059)
Supplement: Supplementary file 1 — Data S1: men70059‐sup‐0001‐Supinfo.docx. [file MEN-26-e70059-s003.docx]

**Non-Invasive Underwater Swab DNA Sampling Illuminates Red Sea Echinoderm Diversity**

Mai Bonomo^1,2^ and Omri Bronstein^1,2^

^1^ George S. Wise Faculty of Life Sciences, School of Zoology, Tel Aviv University, Tel Aviv 6997801, Israel

^2^ The Steinhardt Museum of Natural History, Tel Aviv University, 12 Klausner Street, Tel Aviv, Israel

**Corresponding authors:** Mai Bonomo; Omri Bronstein

Email: [mai.bonomo@gmail.com](http://mai.bonomo@gmail.com) (MB); [bronstein@tauex.tau.ac.il](mailto:bronstein@tauex.tau.ac.il) (OB)

**Supplementary Materials**

**Supplementary Table 1**. A list of all samples used in the current study and their respective metadata (provided Excel spreadsheet).

**Supplementary Table 2.** A list of sequences generated in the present study as well as sequences obtained from GenBank.

**Supplementary Table 3.** Summary of DNA extractions, amplifications and sequencing success rates for Echinoderm samples used in the current study.

**Supplementary Table 4**. Primer sequences and PCR reaction conditions used in the present study.

**Supplementary Table 5**. TapeStation results – Swab vs. Tissue samples.

**Supplementary Table 6.** TapeStation results – Preservation method.

**Supplementary Table 7.** References for distribution in the Red Sea for species of Ophiuroidea, Crinoidea, Echinoidea, and Asteroidea. Sheet 1 shows all Red Sea species and where they were cited. Sheet 2 gives full references of each citation.

**Supplementary Figure 1**. Preparation steps and instructions for the “double tube” method.

**Supplementary Figure 2.** A swab kit prepared using the “double tube” method.

**Supplementary Video 1**. Preparation of the 5 mL PET bottles for sampling.

**Supplementary Video 2**. Underwater swabbing using the 5 mL PET swab and bottle kit.

**Supplementary Table 3.** Summary of DNA extractions, amplifications and sequencing success rates for Echinoderm samples used in the current study.

| **Echinoderm class** | **No. ind. sampled** | **DNA extracted** | **PCR performed** | **PCR success** | **Sent for sequencing** | **Successful sequencing** |
| --- | --- | --- | --- | --- | --- | --- |
| Asteroidea | 51 | 51 | 51 | 51 (100%) | 47 | 44 (93.62%) |
| Crinoidea | 12 | 12 | 12 | 12 (100%) | 10 | 9 (90.00%) |
| Echinoidea | 73 | 61 | 61 | 55 (90.16%) | 41 | 40 (97.56%) |
| Holothuroidea | 138 | 136 | 136 | 111 (81.62%) | 106 | 100 (94.34%) |
| Ophiuroidea | 13 | 13 | 13 | 11 (84.62%) | 10 | 9 (90.00%) |
| **SUM:** | **287** | **273** | **273** | **240** | **214** | **202** |
|  | **Average PCR:** | **87.91%** | **Average sequencing:** | **94.39%** |  |  |

**Supplementary Table 4.** Primer sequences and PCR reaction conditions used in the present study.

*Figure - Let me know your thoughts (rough sketch obviously, will remake in illustrator)*

| ***Primer name*** | ***Primer sequence***  ***(5’-3’)*** | ***Amplicon length*** | ***Annealing temp.*** | ***Reference*** |
| --- | --- | --- | --- | --- |
| ***COIceF*** | ACTGCCCACGCCCTAGTAATGATATTTTTTATGGTNATGCC | 655 bp | 55°C | Hoareau and Boissin, 2010 |
| ***COIceR*** | TCGTGTGTCTACGTCCATTCCTACTGTRAACATRTG |  |  |  |
| ***COIe-F*** | ATAATGATAGGAGGRTTTGG | 650 bp | 50°C | Arndt, 1996 |
| ***COIe-R*** | GCTCGTGTRTCTACRTCCAT |  |  |  |
| ***COI16bf*** | AGCCAGGTCAGTTTCTATCT | 1000 bp | 65°C | Bronstein et al. 2019 |
| ***COIer*** | GCTCGTGTRTCTACRTCCAT |  |  | Zeng and Zhang, 2012 |
| ***LCO1490*** | GGTCAACAAATCATAAAGATATTGG | 710 bp | 51°C | Folmer et al. 1994 |
| ***HCO2198*** | TAAACTTCAGGGTGACCAAAAAATCA |  |  |  |

**Supplementary Table 5.** TapeStation results (as DNA integrity number – DIN) of Swab vs. Tissue, for *Tripneustes gratilla elatensis* and *Echinometra* sp. EZ.

| **Individual** | **Species** | **Sample type** | **Concentration (ng/μL)** | **DIN** |
| --- | --- | --- | --- | --- |
| tg1 | *T. g. elatensis* | Swab | 2.21 | - |
| tg1 | *T. g. elatensis* | Tissue | 99.4 | 6.3 |
| tg2 | *T. g. elatensis* | Swab | 3.19 | 7.7 |
| tg2 | *T. g. elatensis* | Tissue | 130 | 6 |
| tg3 | *T. g. elatensis* | Tissue | 150 | 6 |
| tg3 | *T. g. elatensis* | Swab | 5.97 | 7 |
| tg4 | *T. g. elatensis* | Tissue | 101 | 2.7 |
| tg4 | *T. g. elatensis* | Swab | 3.64 | 8.1 |
| tg5 | *T. g. elatensis* | Tissue | 107 | 4.6 |
| tg5 | *T. g. elatensis* | Swab | 1.67 | - |
| ez6 | *Echinometra* sp. EZ | Tissue | 29.5 | 4.4 |
| ez6 | *Echinometra* sp. EZ | Swab | 2.1 | - |
| ez7 | *Echinometra* sp. EZ | Tissue | 60 | 5.7 |
| ez7 | *Echinometra* sp. EZ | Swab | 3.66 | 2.2 |
| ez8 | *Echinometra* sp. EZ | Tissue | 119 | 4.6 |
| ez8 | *Echinometra* sp. EZ | Swab | 6.17 | 7.2 |
| ez9 | *Echinometra* sp. EZ | Tissue | 132 | 6 |
| ez9 | *Echinometra* sp. EZ | Swab | 4.07 | 6.6 |
| ez10 | *Echinometra* sp. EZ | Tissue | 14.2 | 6 |
| ez10 | *Echinometra* sp. EZ | Swab | 7.37 | 3.4 |

**Supplementary Table 6.** TapeStation results (as DNA integrity number – DIN) of *T. g. elatensis* swab samples based on preservation method.

| **Individual** | **Species** | **Sample Type** | **Concentration (ng/μL)** | **DIN** | **Preservation Medium** | **Temperature** |
| --- | --- | --- | --- | --- | --- | --- |
| t1 | *T. g. elatensis* | Swab | 2.32 | - | RNA Save | Fridge (-20°C) |
| t1 | *T. g. elatensis* | Swab | 18.4 | 6.6 | EtOH 100% | Fridge (-20°C) |
| t2 | *T. g. elatensis* | Swab | 6.84 | 8.3 | RNA Save | Fridge (-20°C) |
| t2 | *T. g. elatensis* | Swab | 24.3 | 6.7 | EtOH 100% | Fridge (-20°C) |
| t3 | *T. g. elatensis* | Swab | 6.04 | 8.3 | RNA Save | Fridge (-20°C) |
| t3 | *T. g. elatensis* | Swab | 15 | 7.1 | EtOH 100% | Fridge (-20°C) |
| t4 | *T. g. elatensis* | Swab | 4.93 | 8.1 | RNA Save | Room temp |
| t4 | *T. g. elatensis* | Swab | 16.2 | 7.7 | EtOH 100% | Room temp |
| t5 | *T. g. elatensis* | Swab | 4.68 | 7 | RNA Save | Room temp |
| t5 | *T. g. elatensis* | Swab | 11.2 | 6.5 | EtOH 100% | Room temp |

**Supplementary Figure 1**. Double-tube preparation (alternative method).

**A.** 2 mL Eppendorf tubes are filled 2/3rds of the way with 100% Ethanol or an alternative preservative. A small piece of Parafilm is folded in half and placed on top of the open tube. Next, a 0.5 mL tube is cut midway. The conical tip is discarded and the top half with the flip lid is slowly pushed into the Parafilm and the open 2 mL tube. The result should be a snug fitting double-cap vial, sealing the ethanol within the 2 mL tube. Tubes should be well labeled in several locations in case of ethanol leakage, and relabeled after the dive.

**B.** Buccal swabs are thicker and can be used for larger smoother surfaces while Nasopharyngeal swabs are thinner and better suited for sampling small organisms. Swabs are often fitted with a break point (usualy 3–8 cm from the swab head). The 3 cm break point are ideal for inserting the swab heads into the test tube, longer break points nessesitate the use of sissors or clippers.

**C.** When back on shore or in a lab, seawater from the inserted half tube is decanted prior to removal and discarding of this piece together with the Parafilm. Thereafter, the 2 mL tube is closed, and stored until extraction.


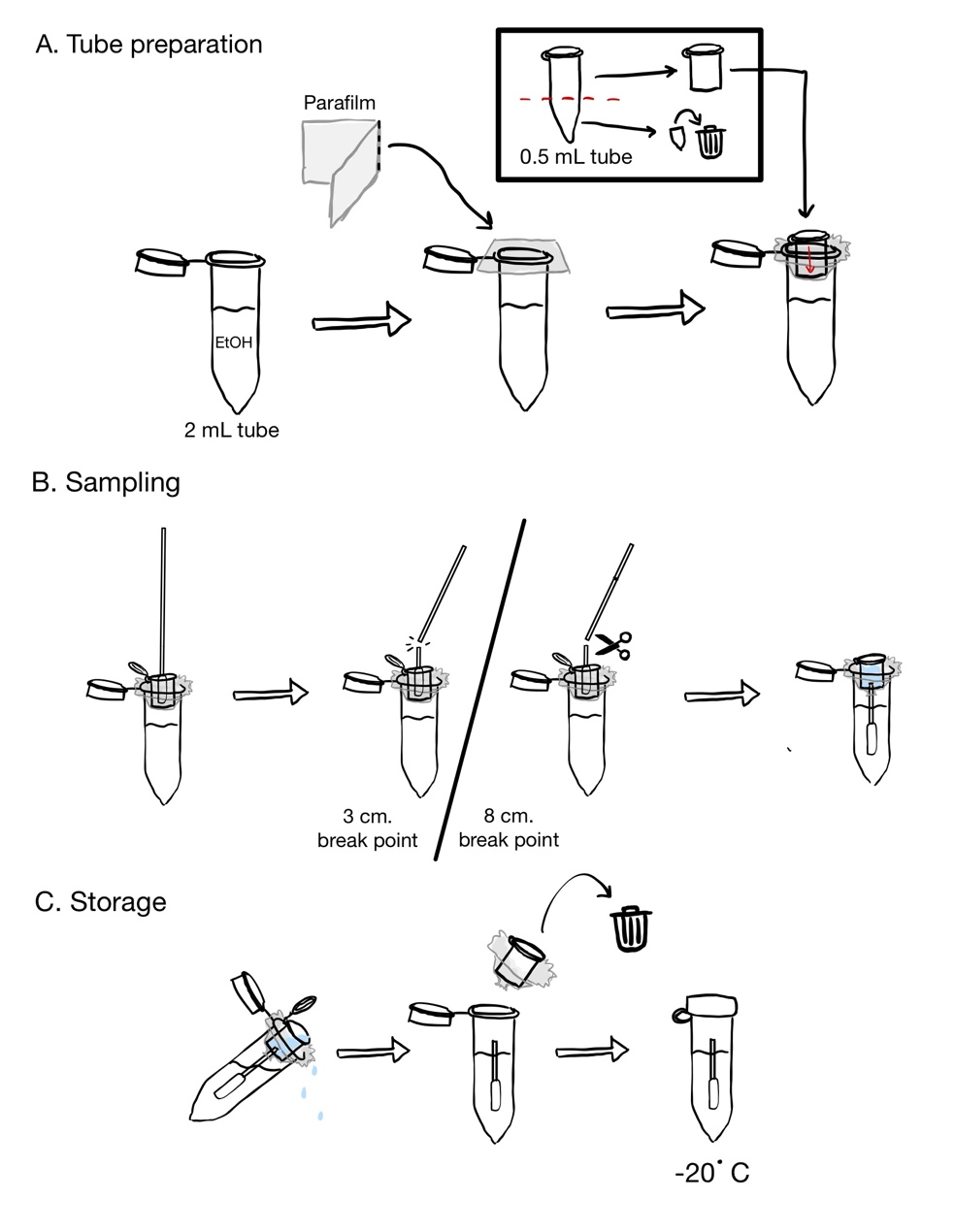


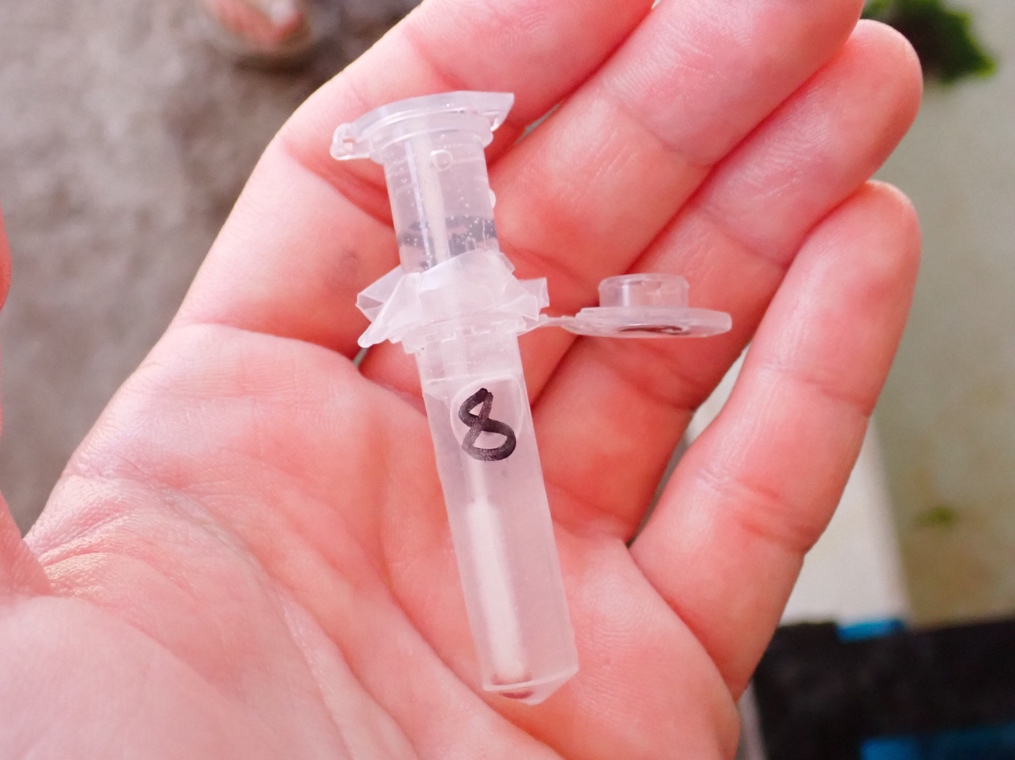


**Supplementary Figure 2 –** A swab collected in a “double-tube”; the top part being filled with seawater, and the bottom with a preservative, with parafilm separating the two spaces.

**References**

Adeli, B., Ghavam Mostafavi, P., & Fatemi, M. R. (2019). Morphological identification and phylogenetic analysis of Asteroidea in the northern coast of the Persian Gulf. *Iranian Journal of Fisheries Sciences*, *19*(6), 3034–3049.

Al-Rshaidat, M. M. D., Snider, A., Rosebraugh, S., Devine, A. M., Devine, T. D., Plaisance, L., Knowlton, N., & Leray, M. (2016). Deep COI sequencing of standardized benthic samples unveils overlooked diversity of Jordanian coral reefs in the Northern Red Sea. *Genome*, *59*(9), 724–737. https://doi.org/10.1139/gen-2015-0208

Alcazar, D. S., & Kochzius, M. (2015). Genetic population structure of the Blue Sea Star *Linckia laevigata* in the Visayas (Philippines). *Journal of the Marine Biological Association of the United Kingdom*, 1–7. https://doi.org/10.1017/s0025315415000971

Boissin, E., Hoareau, T. B., Paulay, G., & Bruggemann, J. H. (2016). Shallow-water reef Ophiuroids (Echinodermata: Ophiuroidea) of Réunion (Mascarene Islands), with biogeographic considerations. *Zootaxa*, *4098*(2). https://doi.org/10.11646/zootaxa.4098.2.4

Bribiesca‐Contreras, G., Solís‐Marín, F. A., Laguarda‐Figueras, A., & Zaldívar‐Riverón, A. (2013). Identification of Echinoderms (Echinodermata) from an anchialine cave in Cozumel Island, Mexico, using DNA barcodes. *Molecular Ecology Resources*, *13*(6), 1137–1145. https://doi.org/10.1111/1755-0998.12098

Bronstein, O., Kroh, A., & Haring, E. (2016). Do genes lie? Mitochondrial capture masks the Red Sea collector urchin’s true identity (Echinodermata: Echinoidea: *Tripneustes*). *Molecular Phylogenetics and Evolution*, *104*, 1–13. https://doi.org/10.1016/j.ympev.2016.07.028

Bronstein, O., Kroh, A., Miskelly, A. D., Smith, S. D., Dworjanyn, S. A., Mos, B., & Byrne, M. (2019). Implications of range overlap in the commercially important pan-tropical sea urchin genus *Tripneustes* (Echinoidea: Toxopneustidae). *Marine Biology*, *166*(3). https://doi.org/10.1007/s00227-019-3478-4

Carletti, A., Cardoso, C., Lobo-Arteaga, J., Sales, S., Juliao, D., Ferreira, I., Chainho, P., Dionísio, M. A., Gaudêncio, M. J., Afonso, C., Lourenço, H., Cancela, M. L., Bandarra, N. M., & Gavaia, P. J. (2022). Antioxidant and anti-inflammatory extracts from sea cucumbers and tunicates induce a pro-osteogenic effect in zebrafish larvae. *Frontiers in Nutrition*, *9*. https://doi.org/10.3389/fnut.2022.888360

Collin, R., Venera-Pontón, D. E., Paulay, G., & Boyle, M. J. (2020a). World travelers: DNA barcoding unmasks the origin of cloning asteroid larvae from the Caribbean. *The Biological Bulletin*, *239*(2), 73–79. https://doi.org/10.1086/710796

Collin, R., Venera‐Pontón, D. E., Driskell, A. C., Macdonald, K. S., Geyer, L. B., Lessios, H. A., & Boyle, M. J. (2020b). DNA barcoding of echinopluteus larvae uncovers cryptic diversity in Neotropical echinoids. *Invertebrate Biology*, *139*(2). https://doi.org/10.1111/ivb.12292

Crandall, E. D., Jones, M. E., Muñoz, M. M., Akinronbi, B., Erdmann, M. V., & Barber, P. H. (2008). Comparative phylogeography of two seastars and their ectosymbionts within the Coral Triangle. *Molecular Ecology*, *17*(24), 5276–5290. https://doi.org/10.1111/j.1365-294x.2008.03995.x

Crandall, E. D., Treml, E. A., Liggins, L., Gleeson, L., Yasuda, N., Barber, P. H., Wörheide, G., & Riginos, C. (2014). Return of the ghosts of dispersal past: Historical spread and contemporary gene flow in the blue sea star *Linckia laevigata*. *Bulletin of Marine Science*, *90*(1), 399–425. https://doi.org/10.5343/bms.2013.1052

Crawford, T. J., & Crawford, B. J. (2007). *Linckia multifora* (Echinodermata: Asteroidea) in Rarotonga, Cook Islands: Reproductive mechanisms and ecophenotypes. *Pacific Science*, *61*(3), 371–381. https://doi.org/10.2984/1534-6188(2007)61[371:lmeair]2.0.co;2

Foo, S. H., Taylor, K. H., Messing, C. G., Rouse, G. W., Tay, T. S., Tan, K. S., & Huang, D. (2021). Assessing the taxonomy of *Heterometra*-like feather stars (Echinodermata: Crinoidea: Himerometroidea) based on morphology and Molecular Data. *Systematics and Biodiversity*, *19*(7), 632–647. https://doi.org/10.1080/14772000.2021.1902418

Galac, M. R., Bosch, I., & Janies, D. A. (2016a). Bacterial communities of Oceanic Sea Star (Asteroidea: Echinodermata) larvae. *Marine Biology*, *163*(7). https://doi.org/10.1007/s00227-016-2938-3

Hemery, L., Roux, M., Ameziane, N., & Eleamue, M. (2013). High-resolution crinoid phyletic inter-relationships derived from molecular data. *Cahiers de Biologie Marine*, *54*(4), 511–523.

Hoareau, T. B., & Boissin, E. (2010). Design of phylum‐specific hybrid primers for DNA barcoding: Addressing the need for efficient coi amplification in the Echinodermata. *Molecular Ecology Resources*, *10*(6), 960–967. https://doi.org/10.1111/j.1755-0998.2010.02848.x

Hoareau, T. B., Boissin, E., Paulay, G., & Bruggemann, J. H. (2013). The southwestern Indian Ocean as a potential marine evolutionary hotspot: Perspectives from comparative phylogeography of reef brittle‐stars. *Journal of Biogeography*, *40*(11), 2167–2179. https://doi.org/10.1111/jbi.12155

Ip, Y. C., Tay, Y. C., Gan, S. X., Ang, H. P., Tun, K., Chou, L. M., Huang, D., & Meier, R. (2019). From Marine Park to future genomic observatory? Enhancing marine biodiversity assessments using a biocode approach. *Biodiversity Data Journal*, *7*. https://doi.org/10.3897/bdj.7.e46833

Jeffery, C. H., Emlet, R. B., & Littlewood, D. T. J. (2003). Phylogeny and evolution of developmental mode in temnopleurid echinoids. *Molecular Phylogenetics and Evolution*, *28*(1), 99–118. https://doi.org/10.1016/s1055-7903(03)00030-7

Kogure, Y., Omori, A., & Fujita, Y. (2023). Taxonomic status of the oreasterid sea star *Bothriaster primigenius* Döderlein, 1916 (Echinodermata, Asteroidea). *Biogeography*, *25*, 13–18. https://doi.org/https://doi.org/10.11358/biogeo.25.13

Lanterbecq, D., Rouse, G. W., & Eeckhaut, I. (2010). Evidence for cospeciation events in the host–symbiont system involving crinoids (Echinodermata) and their obligate associates, the myzostomids (Myzostomida, Annelida). *Molecular Phylogenetics and Evolution*, *54*(2), 357–371. https://doi.org/10.1016/j.ympev.2009.08.011

Lee, T. (2011). A systematic study of Korean Echinoids based on morphology and molecular phylogeny. *Sahmyook University*.

Li, Y., Dong, Y., Xu, Q., Fan, S., Lin, H., Wang, M., & Zhang, X. (2020). Genetic differentiation and evolutionary history of the circumpolar species *Ophiura sarsii* and subspecies *Ophiura sarsii vadicola* (Ophiurida: Ophiuridae). *Continental Shelf Research*, *197*, 104085. https://doi.org/10.1016/j.csr.2020.104085

Lopes, E. M., Pérez‐Portela, R., Paiva, P. C., & Ventura, C. R. (2016). The molecular phylogeny of the Sea Star *Echinaster* (Asteroidea: Echinasteridae) provides insights for genus taxonomy. *Invertebrate Biology*, *135*(3), 235–244. https://doi.org/10.1111/ivb.12135

Okanishi, M., & Fujita, T. (2013). Molecular phylogeny based on increased number of species and genes revealed more robust family-level systematics of the order Euryalida (Echinodermata: Ophiuroidea). *Molecular Phylogenetics and Evolution*, *69*(3), 566–580. https://doi.org/10.1016/j.ympev.2013.07.021

Pappalardo, P., Collins, A. G., Pagenkopp Lohan, K. M., Hanson, K. M., Truskey, S. B., Jaeckle, W., Ames, C. L., Goodheart, J. A., Bush, S. L., Biancani, L. M., Strong, E. E., Vecchione, M., Harasewych, M. G., Reed, K., Lin, C., Hartil, E. C., Whelpley, J., Blumberg, J., Matterson, K., … Osborn, K. J. (2021). The role of taxonomic expertise in interpretation of metabarcoding studies. *ICES Journal of Marine Science*, *78*(9), 3397–3410. https://doi.org/10.1093/icesjms/fsab082

Plaisance, L., Matterson, K., Fabricius, K., Drovetski, S., Meyer, C., & Knowlton, N. (2021). Effects of low pH on the coral reef cryptic invertebrate communities near CO2 Vents in Papua New Guinea. *PLOS ONE*, *16*(12). https://doi.org/10.1371/journal.pone.0258725

Rouse, G. W., Jermiin, L. S., Wilson, N. G., Eeckhaut, I., Lanterbecq, D., Oji, T., Young, C. M., Browning, T., Cisternas, P., Helgen, L. E., Stuckey, M., & Messing, C. G. (2013). Fixed, free, and fixed: The fickle phylogeny of extant crinoidea (Echinodermata) and their Permian–triassic origin. *Molecular Phylogenetics and Evolution*, *66*(1), 161–181. https://doi.org/10.1016/j.ympev.2012.09.018

Sonet, G., Smitz, N., Vangestel, C., & Samyn, Y. (2022). DNA barcoding echinoderms from the East Coast of South Africa. The challenge to maintain DNA data connected with taxonomy. *PLOS ONE*, *17*(10). https://doi.org/10.1371/journal.pone.0270321

Stockley, B., Smith, A. B., Littlewood, T., Lessios, H. A., & Mackenzie‐Dodds, J. A. (2005). Phylogenetic relationships of spatangoid sea urchins (Echinoidea): Taxon sampling density and congruence between morphological and molecular estimates. *Zoologica Scripta*, *34*(5), 447–468. https://doi.org/10.1111/j.1463-6409.2005.00201.x

Summers, M. M., Al-Hakim, I. I., & Rouse, G. W. (2014a). Turbo-taxonomy: 21 new species of Myzostomida (Annelida). *Zootaxa*, *3873*(4). https://doi.org/10.11646/zootaxa.3873.4.1

Summers, M. M., Messing, C. G., & Rouse, G. W. (2014b). Phylogeny of Comatulidae (Echinodermata: Crinoidea: Comatulida): A new classification and an assessment of morphological characters for crinoid taxonomy. *Molecular Phylogenetics and Evolution*, *80*, 319–339. https://doi.org/10.1016/j.ympev.2014.06.030

Summers, M. M., Messing, C. G., & Rouse, G. W. (2017). The genera and species of Comatulidae (Comatulida: Crinoidea): Taxonomic revisions and a molecular and morphological guide. *Zootaxa*, *4268*(2). https://doi.org/10.11646/zootaxa.4268.2.1

Takano, T., Kubo, H., & Obuchi, M. (2022). New records of associations between ectoparasitic snails of the genus *Mucronalia* (Caenogastropoda: Eulimidae) and their ophiuroid hosts from Japan and New Caledonia, with description of a new species. *Plankton and Benthos Research*, *17*(3), 255–262. https://doi.org/10.3800/pbr.17.255

Torrence, K. G., Correia, M. D., & Hoffman, E. A. (2012). Divergent sympatric lineages of the Atlantic and Indian Ocean crinoid *Tropiometra carinata*. *Invertebrate Biology*, *131*(4), 355–365. https://doi.org/10.1111/j.1744-7410.2012.00275.x

Vogler, C., Benzie, J., Lessios, H., Barber, P., & Wörheide, G. (2008). A threat to coral reefs multiplied? Four species of crown-of-thorns starfish. *Biology Letters*, *4*(6), 696–699. https://doi.org/10.1098/rsbl.2008.0454

Wakayama, N., Kiyono, Y., Matsumoto, N., Saitoh, M., & Kanazawa, K. (2019). Effective DNA extraction methods for mitochondrial phylogenomics of the sea urchins. *Zoosymposia*, *15*(1), 192–202. https://doi.org/10.11646/zoosymposia.15.1.20

Ward, R. D., Holmes, B. H., & O’Hara, T. D. (2008). DNA barcoding discriminates echinoderm species. *Molecular Ecology Resources*, *8*(6), 1202–1211. https://doi.org/10.1111/j.1755-0998.2008.02332.x

Waters, J. M., Mark O’Loughlin, P., & Roy, M. S. (2004). Molecular systematics of some Indo-Pacific asterinids (Echinodermata, Asteroidea): Does taxonomy reflect phylogeny? *Molecular Phylogenetics and Evolution*, *30*(3), 872–878. https://doi.org/10.1016/j.ympev.2003.08.019

Williams, S. T. (2000). Species boundaries in the starfish genus *Linckia*. *Marine Biology*, *136*(1), 137–148. https://doi.org/10.1007/s002270050016
